# Moderate water stress in rice induces rhizosheath formation associated with abscisic acid and auxin responses

**DOI:** 10.1093/jxb/eraa021

**Published:** 2020-02-08

**Authors:** Yingjiao Zhang, Huan Du, Yao Gui, Feiyun Xu, Jianping Liu, Jianhua Zhang, Weifeng Xu

**Affiliations:** 1 Center for Plant Water-use and Nutrition Regulation and College of Life Sciences, Joint International Research Laboratory of Water and Nutrient in Crop, Fujian Agriculture and Forestry University, Fuzhou, China; 2 Institute of Oceanography, Minjiang University, Fuzhou, China; 3 Department of Biology, Hong Kong Baptist University, State Key Laboratory of Agrobiotechnology in the Chinese University of Hong Kong, Hong Kong, China; 4 Forschungszentrum Jülich, Germany

**Keywords:** ABA, auxin, moderate water stress, rhizosheath formation, root growth, root hair

## Abstract

The rhizosheath is known to be beneficial for drought resistance in many plants, but the regulation of rhizosheath formation in rice plants is unclear. Here, we investigate rhizosheath formation in different rice varieties and root hair mutants. Our results showed that moderate water stress in rice induced rhizosheath formation. The soil porosity and water content were higher in the rice rhizosheath than in the rice bulk soil under moderate water stress. Additionally, rhizosheath formation in short root hair mutants was lower than in wild-type rice under moderate water stress. Moreover, transcriptomic results indicated that abscisic acid (ABA) and auxin were involved in root and root hair responses in rhizosheath formation. Further, blocking ABA and auxin pathways in wild type and in *rhl1-1,* the shortest root hair mutant, rhizosheath formation and root hair length were significantly decreased under moderate water stress. However, wild type plants maintained a higher root ABA content, root basipetal auxin transport, root hair length, and amount of rhizosheath than did *rhl1-1*. Our results suggest that moderate water stress in rice induces rhizosheath formation by modulating the ABA and auxin responses to regulate root and root hair growth, which may be used to breed rice varieties resistant to drought.

## Teaser

Moderate water stress in rice induces rhizosheath formation by modulating the abscisic acid and auxin responses to regulate root and root hair growth.

## Introduction

The combined impact of decreased water availability and rapid human population growth threatens future food security (Caine *et al.*, 2019). Thus, there is a need to identify beneficial traits of crop roots that enhance water-use capacities to increase yields under regular or reduced water input (Thorup-Kristensen & Kirkegaard, 2016). Moderate water stress (MWS) is a promising approach to reduce irrigation input and maintain crop yield (Xu *et al.*, 2013; Song *et al.*, 2018; Zhang *et al.*, 2019b). MWS maintains or increases root elongation and the root hair response, which relies predominantly on abscisic acid (ABA) accumulation and auxin transport in the root tip (Xu *et al.*, 2013; Shi *et al.*, 2015). In addition, our previous results showed that MWS increased rhizosheath formation in foxtail millet, an important drought-adaptive trait (Liu *et al.*, 2019).

The rhizosheath has potential with regard to future agricultural sustainability (Goodchild and Myers, 1987; Brown *et al.*, 2017). It was discovered over 100 years ago and is defined as a layer of soil that adheres strongly to the root surface as a consequence of root hair penetration between soil particles and mucilage secretions from roots or microbes that bind the particles together. Rhizosheath formation is impacted by many factors. For example, Haling *et al.* (2010) found that root hairs are integral to rhizosheath production; Watt *et al.* (1993) indicated that root and microbial mucilages contribute to rhizosheath formation; moreover, soil characteristics, including water content, acidity, and texture, are associated with rhizosheath size (Watt *et al.*, 1994; Haling *et al.*, 2014). Additionally, rhizosheath formation has been linked to genetic factors (Delhaize *et al.*, 2012; George *et al.*, 2014).

Rhizosheaths provide a dynamic zone for water and nutrient interchange at the root–soil interface, which can improve drought resistance and can protect roots against other abiotic stresses (Brown *et al.*, 2017). Bristow *et al.* (1985) reported that grass rhizosheaths significantly influenced water uptake. North and Nobel (1997) found that rhizosheaths facilitated water uptake in the sheathed root region and had higher water content and water potential than bulk soil under drought conditions. Moreover, the hydraulic properties of the rhizosphere were impacted by the structure of the pore space around roots (White *et al.*, 2013). Recently, the effect of root-induced compaction on water flow in the rhizosphere has been investigated *in situ* using three-dimensional (3D) non-destructive imaging and mathematical modelling. Schmidt *et al.* (2012) reported that root–soil contact was related to porosity and aggregate size around roots. Daly *et al.* (2015) reported that there were clear differences in the hydraulic properties between the rhizosphere of wheat and bulk soil, and rhizosphere soil was less porous than bulk soil. In contrast, many studies showed that there were more porous masses at the root–soil interface, which surrounds the growing roots of many plant species, than in bulk soil (Helliwell *et al.*, 2017; Koebernick *et al.*, 2017; Rabbi *et al.*, 2018). Beyond this zone of increased porosity, Helliwell *et al.* (2019) found that root penetration mechanisms also lead to an increase in the densification of soil away from the root–soil interface.

Major cereals, including wheat, maize, barley, oats, rye, and sorghum, have rhizosheaths (Duell and Peacock, 1985). Rice (*Oryza sativa* L.) is one of the most important staple cereals worldwide (Zhang *et al.*, 2019a). However, the formation of the rhizosheath and its regulation in rice are unclear. Rice cultivation is specifically water intensive, requiring approximately 2500 liters of water per kilogram of rice produced (Bouman, 2009). Thus, it is necessary to identify beneficial traits, such as rhizosheaths, that can be used to breed rice cultivars with increased drought resistance. Thus, the aims of this study were to evaluate the variation in rhizosheath formation in rice plants, including different rice varieties and short root hair mutants, to determine the roles of root traits, and to determine the molecular regulation in rhizosheath formation in rice plants.

## Materials and methods

### Plant growth conditions and soil materials

All experiments in this study were conducted in a greenhouse. The greenhouse environment was set to a 14/10 h light/dark cycle, a 26/22 °C day/night temperature cycle, 40% relative humidity, and a photosynthetic photon flux density of 300 μmol photons m^−2^ s^−1^. The soil used in this study was collected from a paddy rice field in the town of Huayang, Jiangxi Province, China (115°09′32′′E, 28°32′29′′N). The loamy sand (55.1% sand, 33.0% silt, and 11.9% clay) contained 20.5 g kg^−1^ soil organic carbon, 1.75 g kg^−1^ total nitrogen, 0.65 g kg^−1^ total P, 27.7 g kg^−1^ total potassium, 42.6 mg kg^−1^ Olsen-P and 92.0 mg kg^−1^ exchangeable potassium. Soil was collected from a depth of 0–20 cm, air dried, mixed, and passed through a 4 mm sieve to remove coarse material and vegetative matter. To ensure stable soil structure and avoid soil compaction during wet–dry–rewet cycles, 1.8 kg dry soil for each pot was mixed with water to a final water content of 20%. After the soil and water were mixed, the soil was passed through a 4 mm sieve again to ensure homogeneity and packed into pots (14 cm diameter, 10 cm height) with a bulk density of about 1 g cm^−3^. After this treatment, the soil maintained a stable structure composed of aggregates to closely represent the field soil conditions.

### Rice varieties and genetic material

The lowland rice varieties Nipponbare (Nip) and Dongjing (Dj) and upland rice varieties Gaoshan1 (Up1) and Zhonghan3 (Zh3) were used for the analysis of rhizosheath formation. To investigate the effect of root hairs on rhizosheath formation, lowland rice Kasalath (Ka) and two rice root hair mutants in the Ka background, wild-type (WT), *expa17* (*Osexpa17*, shorter root hair mutant), and *rhl1-1* (*Osrhl1-1*, the shortest root hair mutant), were used. The *expa17* and *rhl1-1* mutants, which were identified in an ethyl methanesulfonate mutant library of the Indica rice cultivar Ka, have shorter root hairs than WT but a similar root hair density (Ding *et al.*, 2009; Yu *et al.*, 2011). Seeds were surface-sterilized using 1.5% (v/v) sodium hypochlorite and rinsed with double-distilled water. Three days after germination in Petri dishes on filter paper in the dark at 30 °C, uniform seedlings were transplanted into pots or nutrient solution.

### Pot experiments and rhizosheath screening

#### Irrigation treatments

Nip seedlings were transplanted into pots. After 7 d, the rice plants were subjected to three irrigation treatments: continuous flooding (CF; 3–5 cm water layer maintained), MWS (watered with 100 ml every 3 d), and severe water stress (SWS; watered with 100 ml every 6 d). Intact seedlings were harvested after 12 d of treatment to assess rhizosheath formation.

#### Different rice varieties in rhizosheath formation

To determine the rhizosheath formation in different rice varieties, Nip, Dj, Up1, and Zh3 were grown in pots under the MWS and CF conditions. Four plants of each variety were harvested after 9 d of treatment at the end of the third MWS cycle to assess rhizosheath formation and plant traits.

#### Different root hair lengths in rhizosheath formation

To assess the effect of root hair length on rhizosheath formation, WT, *expa17* (with shorter root hairs), or *rhl1-1* (with the shortest root hairs) plants were subjected to CF, MWS, MWS with the ABA biosynthetic inhibitor fluridone (FLU), and MWS with the auxin efflux inhibitor 1-naphthylphthalamic acid (NPA) conditions in pots. After 9 d of treatment, four plants of each genotype were analysed for rhizosheath formation and plant traits. WT, *expa17*, and *rhl1-1* were also grown in Kimura nutrient solution as described previously (Xu *et al.*, 2010) to conform root hair length.

#### Rhizosheath screen and plant traits

Pots were disassembled, and roots were systematically shaken to remove the bulk soil, leaving the rhizosheath soil. Root and rhizosheath soils were washed, and the rhizosheath soil and the water used to wash it were collected in a tray and dried at 105 °C for 3 d to determine the dry weight of the rhizosheaths. Root length was measured using an Epson scanner (Epson, Herts, UK) and winRHIZO software (Regent Instruments, Quebec, Canada). The amount of rhizosheath per plant was the total rhizosheath soil dry weight of a root, and the rhizosheath per root length was calculated as the total rhizosheath soil dry weight/total root length. The dry weights of roots and shoots were determined using samples that had been dried at 80 °C for 2 d. Dry weight/irrigation water was calculated as the ratio of the total dry mass produced by whole plant to the total volume of irrigation water used. The total irrigation water in the CF and MWS treatments was 3.7 and 2.3 liters, respectively. SPAD readings were taken with a chlorophyll meter (SPAD-502, Konica Minolta, Tokyo, Japan) and recorded as a mean of 10 measurements for each individual leaf.

### Root hair analysis

The longest crown root was harvested for the measurement of root hairs, from which a 5 mm-long root segment was excised at a distance of one-quarter of the total length of the root below the seed as described previously (Delhaize *et al.*, 2012). Images (JPG file format, each one was approximately 2.5 Mb) were obtained using an SMZ18 stereomicroscope and DS-U3 camera (Nikon, Tokyo, Japan). The length of 10 randomly chosen root hairs per image was measured using ImageJ software (National Institutes of Health, MD, USA). Root hair density was measured using the method of Nestler *et al.* (2016).

### Water content measurement

The crown root with rhizosheaths was used for the measurement of water content. After the root was cleaned, the fresh weight was obtained. Rhizosheath soil and bulk soil were collected, and their fresh weights were determined. The dry weights of the root and soil were obtained after 3 d at 60 °C. The water content was calculated as (fresh weight − dry weight)/fresh weight.

### Micro-computed tomography assessment of porosity

For the micro-computed tomography (µCT) assessment of rhizosheath porosity, Nip and Up1 were grown in plastic pots (7 cm diameter, 8 cm height) in 4 mm sieved soil with a dry bulk density of 1 g cm^−3^. After 15 d, the water content was approximately 20%, and the rice plants were scanned by µCT at 190 kV and 180 µA with a voxel spatial resolution of 50 µm (phoenix v|tome|x m, GE Sensing & Inspection, Wunstorf, Germany), with the acquisition of a total of 1600 projection images over a 360° rotation. Each projection image was the average of three images acquired using a detector exposure time of 500 ms; the total scan time was 42 min. The images were reconstructed using phoenix datos|x reconstruction software (GE Sensing & Inspection). Image sections, 3D-rendered images, and root extraction were performed using VG StudioMax (version 3.2; Volume Graphics GmbH, Heidelberg, Germany). Roots were segmented using an adaptive region growing algorithm. For porosity analysis, we selected 10 root segments at a depth of 3–6 cm from the soil surface, and each root segment had a 1.5 mm-long straight cylinder as described by Rabbi *et al.* (2018). In this method, the potential impacts of the soil surface or soil bottom were excluded. Rabbi *et al.* (2018) designated a 0–3 mm radius of root as the rhizosheath of chickpeas; however, a 0–0.5 mm radius of root bound with soil was clearly established as the rhizosheath and measured in rice using Vernier calipers; thus the pores within a 0–0.5 mm radius of the root were designated the rhizosheath of rice in this study, and the pores within a 0.5–1.5 mm radius and 1.5–2.5 mm radius were also measured. Concentric circles with 0–0.5, 0.5–1.5, and 1.5–2.5 mm radii were drawn around the root using an Ellipse model. To segment pore and soil phases, the VGDefX algorithm was used, which allows for grey value variations.

### RNA isolation

Rice plants of all varieties or genotypes were subjected to the CF and MWS treatments in pots. Three independent biological repeats containing two plants in each sample were made. Roots bound with rhizosheaths (rhizosheath–root) under MWS and without rhizosheaths under CF were harvested, cleaned, and immediately frozen in liquid nitrogen and stored at −80 °C. Total RNA was extracted according to Liu *et al.* (2016). The roots were pulverized in liquid nitrogen and extracted using TRIzol reagent (Invitrogen, CA, USA). The quality and quantity of the total RNA were assessed using the 2100 Bioanalyzer instrument (Agilent Technologies, CA, USA).

### RNA sequencing and data analysis

The RNA samples from the Nip roots and Up1 roots were processed for RNA sequencing (RNA-seq). Sequencing libraries were generated using NEBNext Ultra (NEB, MA, USA) as described previously (Zhang *et al.*, 2016) and sequenced using the BGISEQ-500 sequencer (BGI, Shenzhen, China). The assessment and cleaning of the raw reads obtained from RNA-seq were performed using SOAPnuk (version 1.5.2). The resulting high-quality reads were mapped against the *Oryza_sativa*_Japonica_Group reference genome (IRGSP-1.0) using HISAT2 (version 2.0.4). To determine transcript abundance, values of fragments per kilobase of transcript per million mapped fragments were calculated using Bowtie2 (version 2.2.5). Gene annotations and Gene Ontology (GO) classifications were obtained from IRGSP-1.0 and GO (http://www.geneontology.org/). Hierarchical clustering analyses were executed using the heatmap.2 function in R with the gplots package. GO enrichment analysis was carried out using Database for Annotation, Visualization and Integrated Discovery (DAVID, https://david.ncifcrf.gov/) with GOTERM_BP_FAT term as previously reported (Clancy *et al.*, 2012).

### Determination of endogenous ABA concentration and assay of auxin transport

To further understand whether ABA and auxin affect rhizosheath formation, root ABA content and root basipetal auxin transport were measured in the WT rice and short root hair mutant *rhl1-1* under MWS, MWS with fluridone (an ABA biosynthetic inhibitor, 10 µM; Xu *et al.*, 2013) and MWS with NPA (an auxin efflux inhibitor, 10 µM; Xu *et al.*, 2013) conditions. The endogenous ABA concentration of the roots was measured using the radioimmunoassay method as described by Xu *et al.* (2013). Root samples (0.2 g) were placed in 1 ml of distilled water, homogenized, and shaken at 4 °C overnight. The homogenates were centrifuged for 10 min at 12 000 *g* in 4 °C. The supernatant was used for the ABA assay. The phosphate buffer (pH 6.0, 200 µl), diluted antibody (Mac 252) solution (100 µl), [^3^H]ABA (8000 cpm) solution (100 µl), and crude extract (50 µl) were mixed and then incubated at 4 °C for 45 min. The bound radioactivity was measured in 50% saturated (NH_4_)_2_SO_4_-precipitated pellets with a liquid scintillation counter. Root basipetal auxin transport was analysed as described by Li *et al.* (2011). One per cent (w/v) agar blocks, which contained 100 nM ^3^H[IAA], were placed in contact with the root tip for 5 h in the dark, and the apical 3 mm sections of the roots were excised for radioactivity counting.

### RT-qPCR

For reverse-transcription quantitative polymerase chain reaction (RT-qPCR) analysis, total RNA (1 μg) was reverse-transcribed into cDNA using the PrimeScrip RT Reagent Kit with gDNA Eraser (TaKaRa, Dalian, China) according to the manufacturer’s instructions. RT-qPCR was performed as described previously (Shi *et al.*, 2017). The transcript levels were normalized to that of *ACTIN1* as an endogenous control. The primers used are listed in Supplementary Table S1. Each of three biological replicates was represented by three technical replicates.

### Statistical analysis

Root trait data (root length, root hair length and density, root:shoot ratio, root weight, plant dry weight/irrigation water ratio, and rhizosheath weight), hormone data (endogenous ABA content of roots and root basipetal auxin transport) and soil trait data (water content and µCT porosity) were analysed with one-way ANOVA at *P*<0.05 using SPSS (version 17.0). Data were checked for normality using a homogeneity of variance test. Duncan’s test was used for *post hoc* multiple comparisons when group sizes were equal, and Tamhane’s T2 test was used for *post hoc* multiple comparisons when group sizes were unequal. Differentially expressed genes (DEGs) were estimated using DEGseq (version 1.18.0) in the Bioconductor package. Genes with a 2-fold difference and an adjusted *P*-value of ≤0.001 were deemed significant. RT-qPCR data were analysed with Student’s *t*-test (*P*<0.05) in SPSS.

## Results

### Dry weight/irrigation water and plant responses in different rice varieties during MWS and CF irrigation

**Fig. 1.**
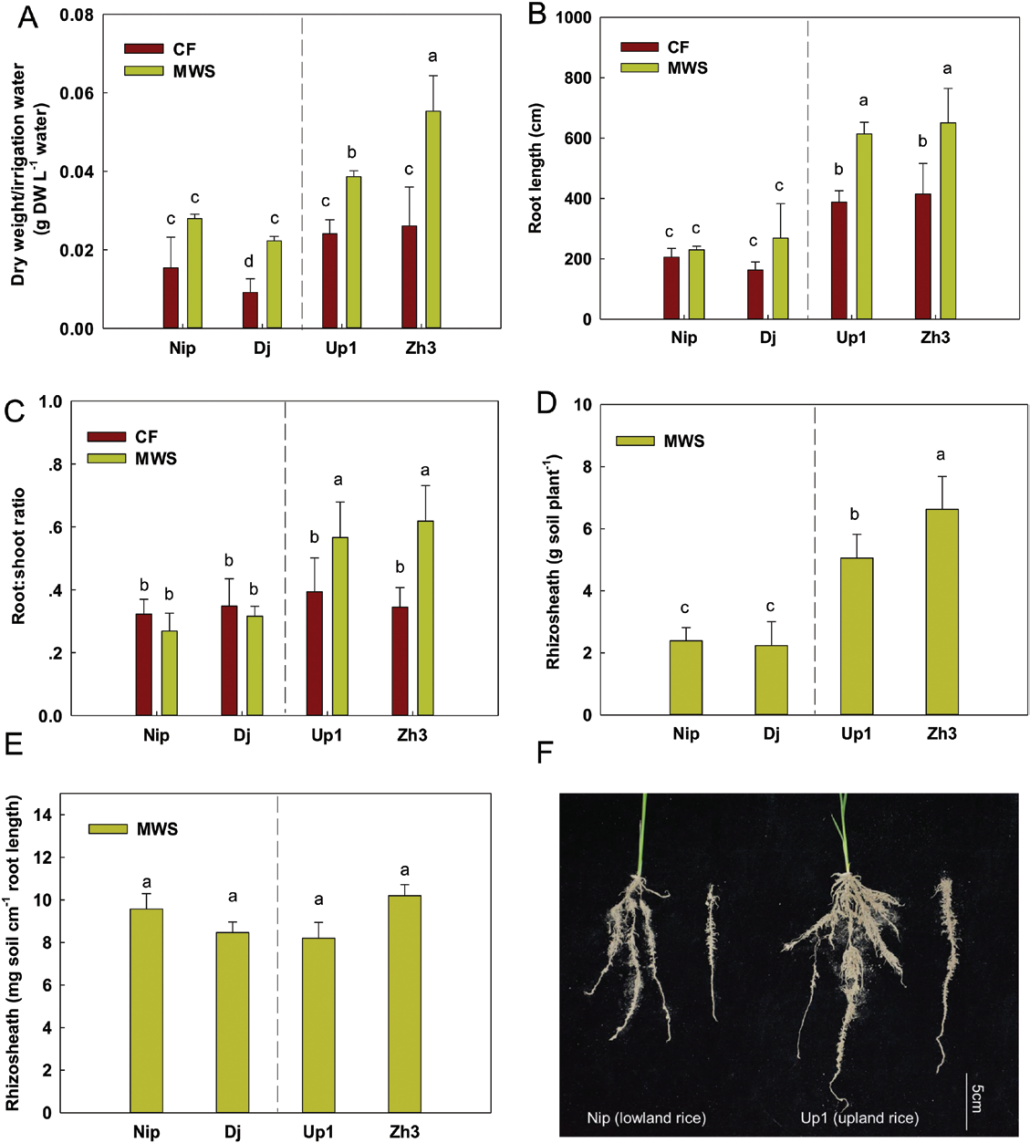
Dry weight/irrigation water, root traits, and rhizosheaths of four rice varieties (Nip, Dj, Up1, and Zh3) under moderate water stress (MWS) and continuous flooding (CF) conditions. (A–C) The whole plant dry weight/irrigation water (A), root length (B) and root:shoot ratio (C) under the MWS and CF conditions in pots. (D, E) The weight of rhizosheath per plant (D) and per centimetre of root length (E) under the MWS condition in pots. (F) Phenotype of the rhizosheaths in Nip and Up1. Data are the means ±SE of four replicates. Bars with different letters were significantly different at *P*<0.05. (This figure is available in color at *JXB* online.)

Compared with the CF condition, MWS did not affect the root length of Nip, while SWS decreased the root length of Nip (*P*<0.05; Supplementary Fig. S1). Thus, we compared the whole plant dry weight/irrigation water and root traits of Nip and Dj with those of Up1 and Zh3 under the MWS and CF conditions. The whole plant dry weight/irrigation water was significantly increased under MWS compared with CF (*P*<0.05) for all the varieties with the exception of Nip, for which it was slightly increased (*P*>0.05; Fig. 1A). Under the CF condition, the whole plant dry weight/irrigation water of Up1 and Zh3 was slightly greater than those of Nip and Dj (*P*<0.05); however, the difference was markedly greater under MWS than under CF conditions (*P*<0.05). The root length and root:shoot ratio of Up1 and Zh3 but not Nip and Dj were significantly increased under MWS (*P*<0.05; Fig. 1B and C). All rice varieties formed rhizosheaths under MWS, but not under CF. Under MWS, the rhizosheath weight per plant was 2- to 3-fold higher in Up1 and Zh3 than in Nip and Dj (Fig. 1D, F); however, there was no significant difference in the rhizosheath weight per centimeter of root length (*P*=0.140; Fig. 1E). Water treatments did not significantly change the plant height and chlorophyll content in the four rice varieties (Supplementary Fig. S2). These results showed that MWS induced rhizosheath formation in the four rice varieties, and the rhizosheath weight per plant was larger in Up1 and Zh3 than in Nip and Dj. In addition, MWS greatly enhanced the dry weight/irrigation water, root length, and root:shoot ratio of Up1 and Zh3.

### Root hair response to moderate water stress in soil

We measured the root hair length and root hair density in soil-grown rice plants. The average root hair length was significantly increased under MWS compared with CF (*P*<0.05), while the root hair density was stable under these two irrigation treatments in Nip, Dj, Up1, and Zh3 (Supplementary Fig. S3). The average root hair length was longer in Nip and Up1 than in Zh3 and Dj under identical irrigation treatments (*P*<0.05; Supplementary Fig. S3A). The root hair density was similar in Nip, Up1, and Zh3 under both CF and MWS conditions. Dj had a shorter root hair density than Up1 and Zh3 under CF or MWS conditions (*P*<0.05; Supplementary Fig. S3B).

### Water content of the root, rhizosheath, and bulk soil under moderate water stress conditions

The water content was higher in the roots but lower in the bulk soil in Up1 and Zh3 (root: both 88.59%; bulk soil: 15.26% and 15.44%, respectively; Table 1) than in Nip and Dj (root: 86.29% and 86.16%, respectively; bulk soil: 17.74% and 18.00%, respectively). The water content was 1.95- to 3.44-fold greater in the rhizosheath (86.16–88.59%) than in the bulk soil (35.42–53.06%, Table 1). The water content in the rhizosheath was higher in Up1 and Zh3 (44.01% and 53.06%, respectively) than in Nip and Dj (37.12% and 35.42%, respectively). These results showed that rhizosheaths could hold water for plant growth and that the rhizosheaths of Up1 and Zh3 had higher water contents than those of Nip and Dj.

**Table 1.** Water contents of the root, rhizosheath, and bulk soil of the four rice varieties (Nip, Dj, Up1, and Zh3) under moderate water stress conditions

Variety	Root (%)	Rhizosheath (%)	Bulk soil (%)	Rhizosheath/bulk soil
Nip	86.29±0.41 b	37.12±3.60 c	17.74±0.94 a	2.09±0.14 c
Dj	86.16±0.87 b	35.42±2.41 c	18.61±0.45 a	1.95±0.06 c
Up1	88.59±0.06 a	44.01±1.71 b	15.26±0.08 b	2.91±0.07 b
Zh3	88.59±1.09 a	53.06±2.39 a	15.44±0.31 b	3.44±0.14 a

Different letters indicate statistical significance at the *P*<0.05 level within the same column. Data are the means ±standard error of four replicates.

### Rhizosheath porosity under moderate water stress conditions

**Fig. 2.**
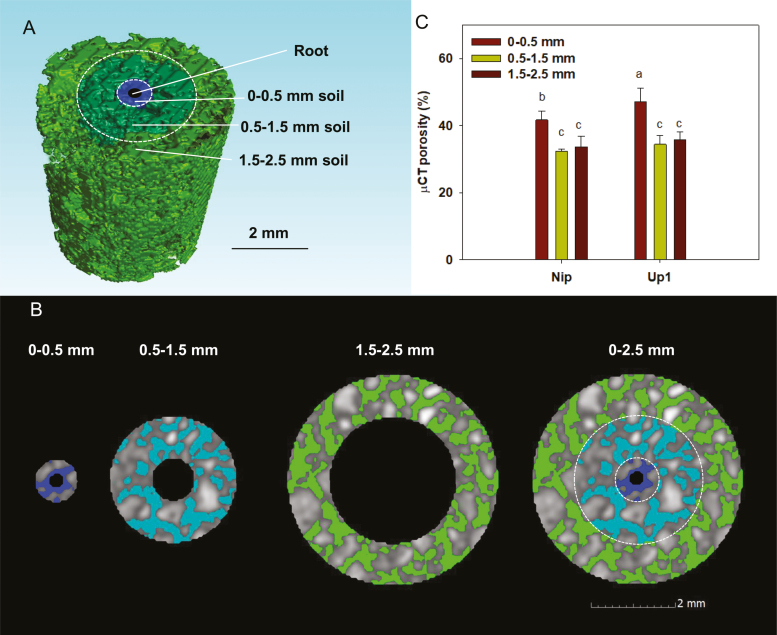
Porosity of the rhizosheath (0–0.5 mm radius concentric circle) and other soil (0.5–1.5 mm radius and 1.5–2.5 mm radius concentric circles) of two rice varieties (Nip and Up1) under the MWS condition. (A) Three-dimensional soil pores around the root. The 0.5 mm radius concentric circle around the root clearly established the rhizosheath; thus, the 0.5 mm radius concentric circle was defined as the rhizosheath. Soil pores in the 0.5–1.5 and 1.5–2.5 mm radius concentric circles were also analysed. (B) Division of each cylinder into three concentric rings around the root. Extracted root: hole in the middle of the 0–0.5 mm radius concentric circle; soil: grey; pores: blue and green. (C) Porosity of the rhizosheath (0–0.5 mm) and other soil (0.5–1.5 and 1.5–2.5 mm). Bars with different letters were significantly different at *P*<0.05.

The 0–0.5 mm radius of soil (rhizosheath) around the roots of Nip and Up1 exhibited greater µCT porosity than the 0.5–1.5 mm radius and 1.5–2.5 mm radius of soil (*P*<0.05; Fig. 2). The µCT porosity of the rhizosheath, approximately the 0–0.5 mm radius of soil, was significantly greater in Up1 than in Nip (*P*<0.05; Fig. 2).

### Dry weight/irrigation water and root traits in root hair mutants during MWS and CF irrigation

**Fig. 3.**
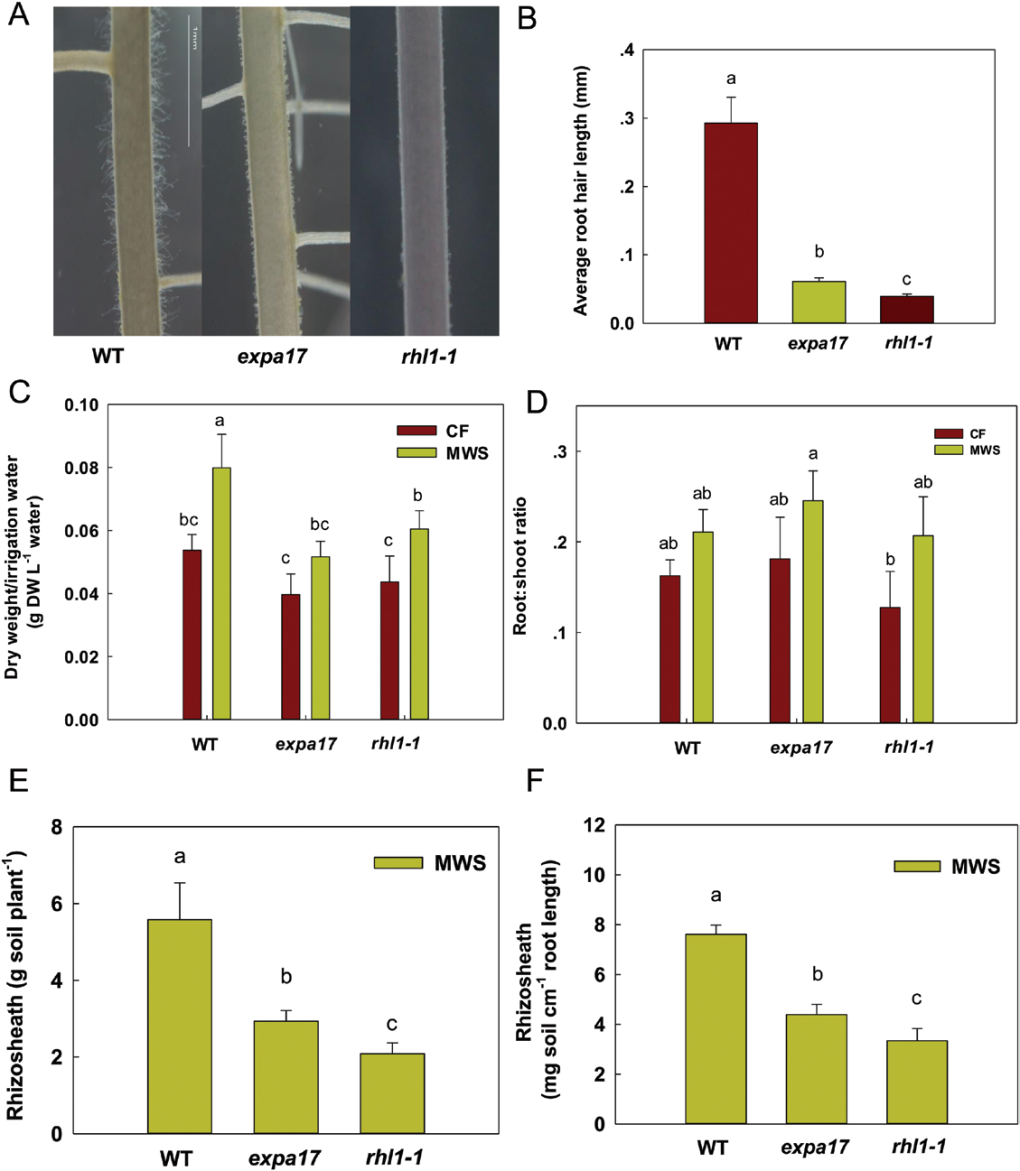
Dry weight/irrigation water, root traits and rhizosheaths of root-hair mutants under moderate water stress (MWS) and continuous flooding (CF) conditions. (A) Photographs of the roots of wild-type (WT, Ka) rice, *expa17* (shorter root hair mutant), and *rhl1-1* (the shortest root hair mutant) in nutrient solution. Scale bar, 1 mm. (B) Average root-hair length of WT, *expa17*, and *rhl1-1* in nutrient solution. (C, D) The whole plant dry weight/irrigation water (C) and root:shoot ratio (D) of WT, *expa17*, and *rhl1-1* under the MWS and CF conditions in pots. (E, F) Total weights of rhizosheaths per plant (E) and per centimetre of root length (F) in WT, *expa17*, and *rhl1-1* under the MWS condition in pots. Data are the means ±SE of four replicates. Bars with different letters were significantly different at *P*<0.05. (This figure is available in color at *JXB* online.)

The root hair lengths of WT rice (~300 µm root hair length), *expa17* (shorter root hair mutant: ~60 µm root hair length), and *rhl1-1* (the shortest root hair mutant: ~40 µm root hair length) were confirmed in nutrient solution (Fig. 3A, B). The WT had higher dry weight/irrigation water under MWS conditions than had *expa17* or *rhl1-1* (*P*<0.05; Fig. 3C). Similar to Nip and Dj, the root:shoot ratios of WT, *expa17*, and *rhl1-1* were similar under the MWS and CF conditions (Fig. 3D). All mutants and WT formed rhizosheaths under MWS conditions but not under CF conditions. The weight of the rhizosheath per plant and per centimeter of root length was greater in the WT than in *expa17* and *rhl1-1* (*P*<0.05; Fig. 3E, F). Previous studies have shown that except for root hair length, there are no significant differences in the root parameters (for example, root length) of WT, *expa17*, and *rhl1-1* (Ding *et al.*, 2009; Yu *et al.*, 2011). Here, root lengths were also measured under MWS, and the results showed that root lengths were similar among WT, *expa17*, and *rhl1-1* (*P*>0.05; Supplementary Fig. S4A). These results showed that root hair length determined rhizosheath formation in WT and root hair mutants.

### RNA-seq global analysis

RNA-seq results showed that over 1 G of clean bases were obtained from each sample (Supplementary Table S2). Over 93% of the clean reads were aligned to the reference genome (Supplementary Table S2). A large proportion of DEGs, up to 1741 DEGs, were found in rhizosheath roots compared with roots without rhizosheaths in both Nip and Up1; there were 1784 and 1421 DEGs only found in Nip rice and Up1 rice, respectively (Supplementary Fig. S5A, B). GO enrichment analysis of these 1741, 1784, and 1421 DEGs is also shown in Supplementary Dataset S1. In Up1, there were 3162 DEGs, comprising 1684 up-regulated and 1478 down-regulated genes, under MWS compared with CF. In Nip, there were 3525 DEGs, comprising 2164 up-regulated and 1361 down-regulated genes, under MWS compared with CF. The number of up-regulated genes in rhizosheath roots under MWS was greater in Nip than in Up1. The expression levels of 11 DEGs (nine up-regulated and two down-regulated) were validated by RT-qPCR (Supplementary Table S3).

### DEGs related to ABA, auxin, and root and root hair growth

**Fig. 4.**
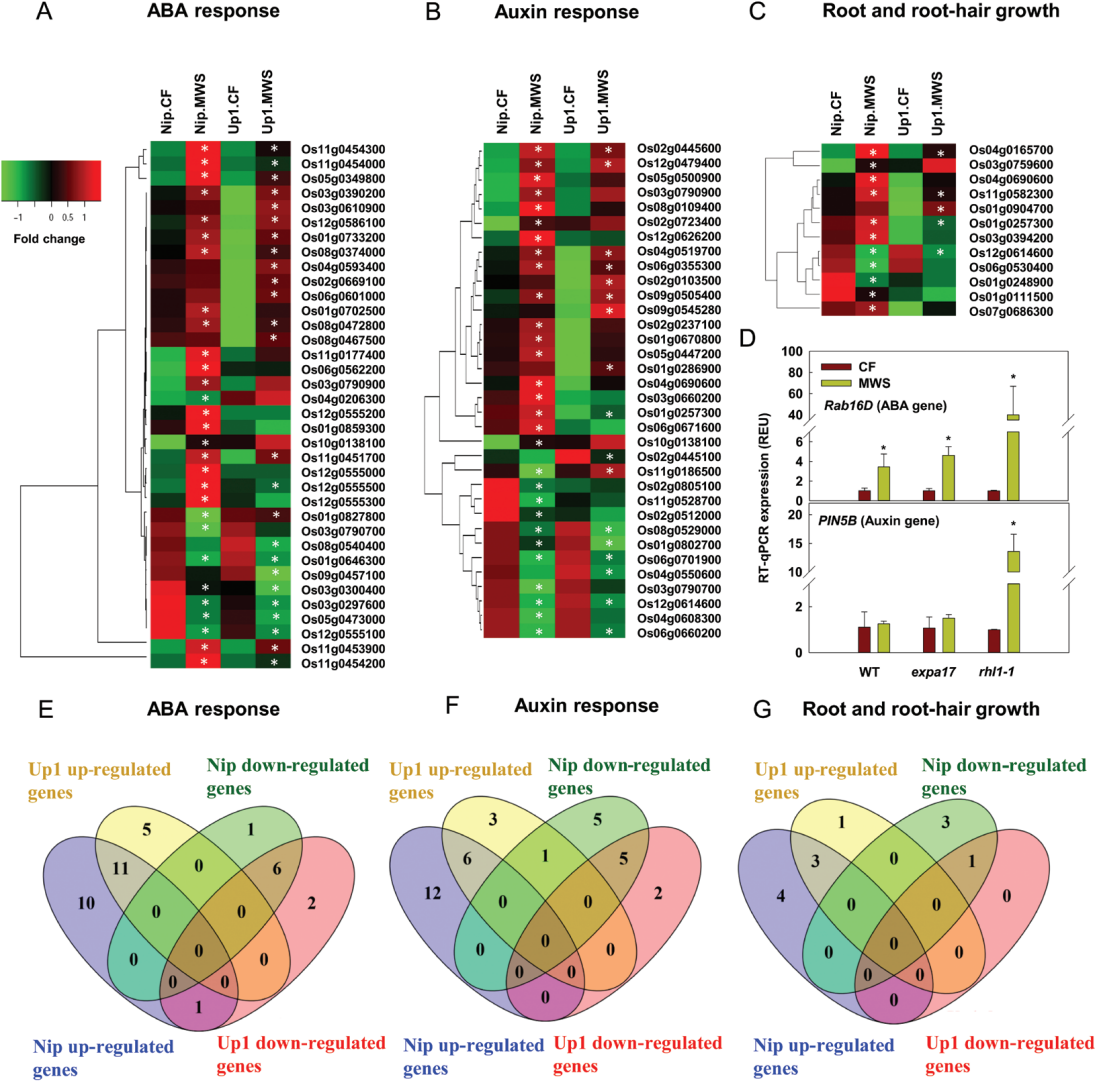
Differentially expressed genes (DEGs) related to ABA, auxin, and root and root-hair growth in two rice varieties (Nip and Up1) under moderate water stress (MWS; with rhizosheath) and continuous flooding (CF; without rhizosheath) conditions. (A–C) Heatmap of DEGs involved in the ABA response (A), auxin response (B) and root and root hair growth (C). Asterisks indicate corrected *P*-value<0.001 and log_2_ (fold change)>1. (D) Expression of the ABA gene *Rab 16D* and the auxin gene *PIN5B* in wild-type (WT, Ka) rice, *expa17* (shorter root hair mutant), and *rhl1-1* (the shortest root hair mutant) by RT-qPCR. Relative expression levels were normalized to that of *ACTIN1* (100 relative expression units (REU)). *P<0.05. (E–G) Venn diagram of DEGs involved in the ABA response (E), auxin response (F), and root and root hair growth (G). (This figure is available in color at *JXB* online.)

The DEGs were functionally classified using GO terms. In the biological process category of the GO terms, the processes of signaling and growth were largely enriched in both Up1 and Nip (Supplementary Fig. S5C, D). ABA is an important signaling factor during water stress. There were 29 DEGs in Nip and 25 DEGs in Up1 that related to the ABA signaling pathway, biosynthesis, or response (Fig. 4A, E; Supplementary Datasets S2, S3). For example, several stress-responsive dehydrins, including *Rab 16B* (Os11g0454200), *Rab 16C* (Os11g0454000), *Rab 16D* (Os11g0453900), *Rab 17* (Os11g0451700), and *Rab 21* (Os11g0454300), were up-regulated under the MWS condition in Up1 and Nip. The expression of *Rab 16D* was also assessed in Ka (WT), *expa17*, and *rhl1-1* by RT-qPCR (Fig. 4D). MWS induced the expression of *Rab 16D* in all of the rice varieties but to the greatest extent in *rhl1-1*. Moreover, the serine/threonine protein kinase genes *SAPK1* (Os03g0390200) and *SAPK9* (Os12g0586100), which belong to the sucrose non-fermenting 1-related kinase 2 family, were up-regulated more than 2-fold under the MWS condition in the two rice varieties (Fig. 4A). *ABA8ox2* (Os08g0472800), related to ABA catabolism, was up-regulated 2-fold under MWS compared with CF in both Up1 and Nip (Fig. 4A). *Emp1* (Os05g0349800), encoding embryonic abundant protein 1, which has been reported to accumulate under ABA treatment, was up-regulated 99- and 93-fold in Up1 and Nip, respectively, under MWS conditions (Fig. 4A). The genes encoding the ABA receptor PYL4 (Os03g0297600, Os05g0473000, and Os01g0827800) were down-regulated under MWS compared with CF in Up1 and Nip (Fig. 4A). DEGs related to auxin biosynthesis, signaling pathways, or responses were also enriched (29 DEGs in Nip and 17 DEGs in Up1; Fig. 4B, F; Supplementary Datasets S2, S3). For example, in Up1, MWS induced the expression of *PROBABLE AUXIN EFFLUX CARRIER COMPONENT 5B* (*PIN5B*, Os09g0505400) and the auxin-responsive genes *AUXIN RESPONSE FACTOR 10* and *24-LIKE* (*ARF10*, Os04g0519700 and *ARF24*, Os12g0479400), *Gretchen Hagen 3.12* (*GH3.12*, Os11g0186500), *INDOLE-3-ACETIC ACID INDUCIBLE 2* (*IAA22*, LOC_Os06g24850), *SMALL AUXIN UP RNA 39* (*SAUR39*, Os09g0545280), and *SMALL AUXIN UP RNA 50* (*SAUR50*, Os02g0445600). In Nip, the following auxin-related genes were induced: *PIN5B*, *ARF2* (Os05g0447200), *ARF10* (Os04g0519700), *ARF24* (Os12g0479400), *ARF 2-LIKE* (Os01g0670800), *ARF 13* (Os04g0690600), *GH3.4* (Os05g0500900), *INDOLE-3-ACETIC ACID INDUCIBLE 8-LIKE* (*IAA8*, Os02g0723400), *IAA22* (LOC_Os06g24850), and *IAA25* (Os08g0109400, Fig. 4B). However, the expression levels of *AUXIN EFFLUX CARRIER COMPONENT 2-LIKE* (*PIN2*, Os06g0660200), *PROBABLE AUXIN EFFLUX CARRIER COMPONENT 5C* (*PIN4*, Os08g0529000), and *AUXIN-INDUCED PROTEIN 10A5* (Os06g0701900) were down-regulated under the MWS condition in both Up1 and Nip (Fig. 4B). The RT-qPCR results also showed that the expression of *PIN5B* was significantly lower in the roots of Ka and *expa17* than in the roots of *rhl1-1* (Fig. 4D). DEGs related to root growth, including root development and root hair-cell development, were also identified (11 DEGs in Nip and four DEGs in Up1, Fig. 4C, G; Supplementary Datasets S2, S3). For example, *CYSTEINE SYNTHASE* (Os04g0165700) and *RHD3* (Os11g0582300), which encode proteins that regulate the root hair response, were up-regulated under the MWS condition compared with the CF condition in the two rice varieties (Up1 and Nip). These results showed that the ABA, auxin, and root and root hair growth pathways were regulated under MWS.

### Rhizosheath formation related to ABA and auxin responses under moderate water stress

**Fig. 5.**
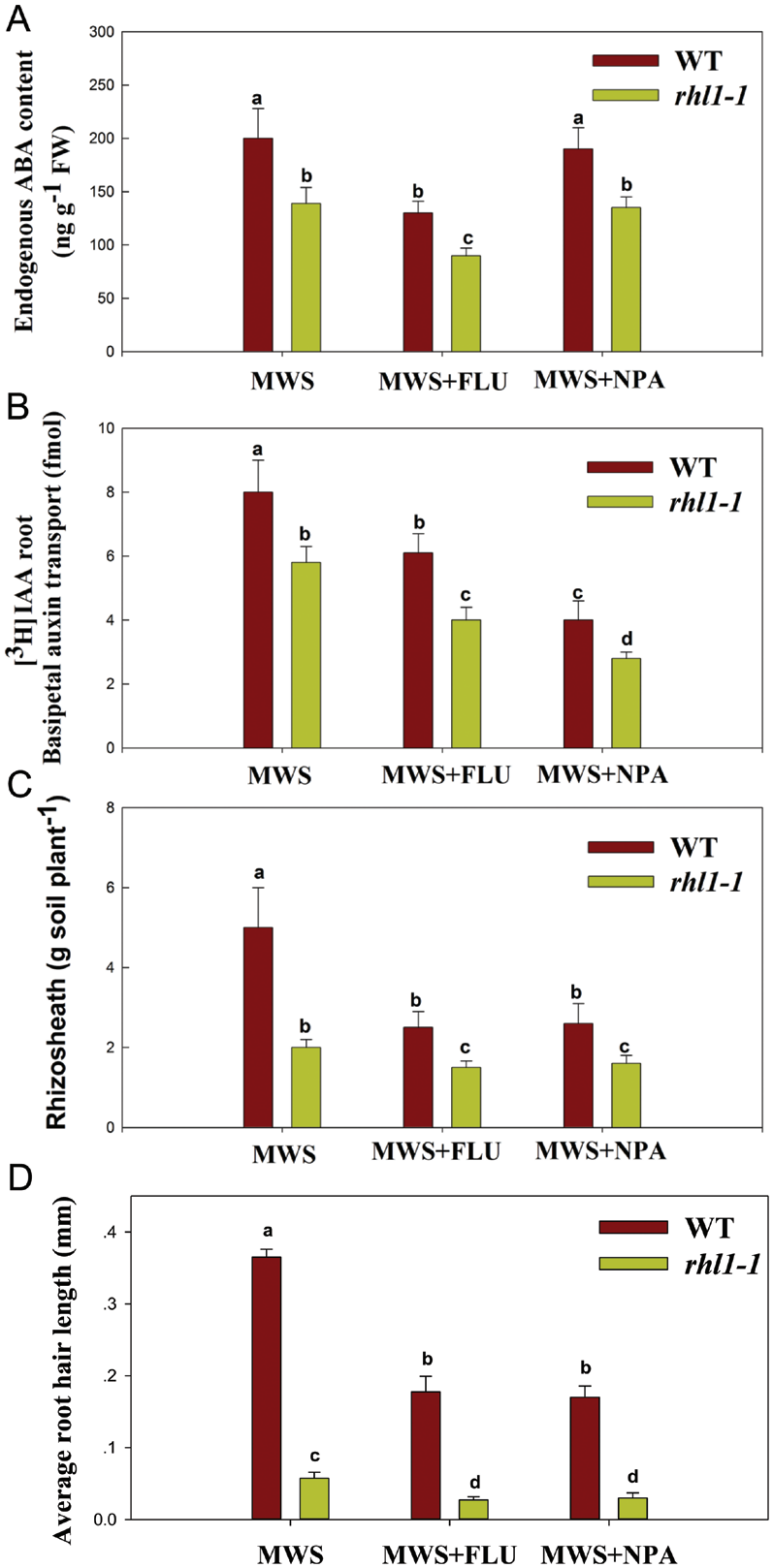
Effect of ABA and auxin responses on rhizosheath formation in wild-type (WT, Ka) rice and the shortest root hair mutant, *rhl1-1*, under moderate water stress (MWS), MWS with fluridone (FLU, an ABA biosynthetic inhibitor), and MWS with NPA (an auxin efflux inhibitor) conditions. (A) Endogenous ABA content of roots, (B) root basipetal auxin transport, (C) amount of rhizosheath per plant, and (D) average root hair length. Data are the means ±SE of four replicates. Bars with different letters were significantly different at *P*<0.05. (This figure is available in color at *JXB* online.)

As shown in Fig. 5, under MWS, the root endogenous ABA content, root basipetal auxin transport, average root hair length, and amount of rhizosheath per plant were significantly higher in WT than in *rhl1-1*. Under MWS with fluridone, the root endogenous ABA content, root basipetal auxin transport, average root hair length, and amount of rhizosheath per plant decreased in both WT and *rhl1-1*; however, WT had a higher root endogenous ABA content, root basipetal auxin transport, average root hair length, and amount of rhizosheath per plant than *rhl1-1*. Under MWS with NPA, NPA significantly suppressed root basipetal auxin transport, average root hair length, and the amount of rhizosheath per plant in both WT and *rhl1-1*, while WT had a higher root basipetal auxin transport, average root hair length, and amount of rhizosheath per plant than *rhl1-1*. However, NPA did not influence the endogenous ABA content of roots in either WT or *rhl1-1*. The root fresh weights, root dry weights, water contents of the roots, and root lengths of WT and *rhl1-1* were not significantly different under different treatments (Supplementary Fig. S4). Because the root length was not significantly changed among the different treatments, the distinction of rhizosheath per plant under different treatments was mainly defined by root hair length (Fig. 5). These results showed that ABA modulation and the auxin pathway may play important roles in rhizosheath formation.

## Discussion

Traditional rice irrigation involves CF to ensure an abundant supply. In rice fields, other than transpiration, water usage includes evapotranspiration, seepage, and percolation. Under CF irrigation, outflows, including seepage and percolation, are high, which explains the 3- to 5-fold higher water requirements of rice compared with other grain crops, such as wheat, barley, and maize (Bouman, 2009). Therefore, a means of saving water for rice production while maintaining its yield is very important. MWS is a water-saving irrigation method for rice that can decrease water outflows and maintain grain yield (Yang *et al.*, 2017). In this study, we found that MWS induced rhizosheath formation in all four rice varieties (Fig. 1).

Soil porosity in the rhizosheath or rhizosphere influences hydraulic processes (Helliwell et al., 2017, 2019; Rabbi *et al.*, 2018). Helliwell *et al.* (2019) showed that new root proliferation may exploit existing pore channels and fissures in soil. Thus, it is possible that the increase in the pores in the rhizosheath may be related to aerial tissue development in rice plants. Previous research using root analogs showed decreased porosity at the root surface (Aravena *et al.*, 2011). However, with the use of µCT to observe the soil structure *in situ*, an increase in porosity at the root surface was found in many studies in real root systems (Helliwell et al., 2017, 2019; Rabbi *et al.*, 2018). To detect the dynamic porosity in soil from the root to the bulk soil, Helliwell et al. (2017, 2019) found that there was an increase in porosity at the root surface to a distance of approximately 0.5 mm, which subsequently declined with distance from the root. A similar result was also found in the study of Rabbi *et al.* (2018), in which the porosity in the rhizosheath (0–3 mm radius of the root) was higher than that of the bulk soil (3–6 mm radius of the root) in drought-tolerant chickpea. In our study, the µCT porosity of the rhizosheath (0–0.5 mm radius of soil around roots) was higher than that of the soil with a 0.5–2.5 mm radius from the roots in Up1 and Nip (Fig. 2), using a method similar to Rabbi *et al.* (2018). Further, the rhizosheath of Up1 had a higher µCT porosity than that of Nip (Fig. 2). In addition, the water content of the rhizosheath was higher than that of the bulk soil in different rice varieties under MWS, and the water content in the rhizosheath of Up1 was higher than that in Nip, but the water content in the bulk soil of Up1 was lower than that of Nip (Table 1). From these results, it was inferred that the rhizosheath can retain relatively more water with its increased porosity and may increase water uptake from bulk soil to reduce the water stress experienced by the roots under MWS. Thus, the rhizosheath is an important root trait for water use in rice production under MWS.

Rhizosheath formation is related to root hair development, which is sensitive to water stress (Haling *et al.*, 2010). However, conflicting results have also been reported for some crops. For example, Delhaize *et al.* (2012) reported a strong correlation between the rhizosheath size and root hair length of wheat in acidic soil; George *et al.* (2014) indicated a weak relationship between root hair length and rhizosheath size for a range of genotypes; and Pang *et al.* (2017) found no correlation between rhizosheath weight and root hair length in 100 chickpea (*Cicer arietinum L.*) genotypes. In this study, we also found no direct association between root hair length and rhizosheath weight under the MWS condition in four rice varieties (Fig. 1E; Supplementary Fig. S3). It was inferred that different rice varieties had different root hair lengths and densities, root diameters, mucilage production, and relationships with soil microorganisms. All of these different root traits in rice varieties could influence rhizosheath formation. However, the short root hair mutants and WT had similar genetic backgrounds and were used to further assess the relationship between root hair length and rhizosheath formation. The results showed that *expa17* (shorter root hairs) had a significantly smaller rhizosheath per centimeter of root length than WT (normal root hair length) but a larger rhizosheath per centimeter of root length than *rhl1-1* (the shortest root hairs, almost hairless, Fig. 3B, F), suggesting that root hair length greatly influences the rhizosheath weight per centimeter of root length based on similar genetic backgrounds.

Although the rhizosheath weight per centimeter of root length was similar in Nip, Dj, Up1, and Zh3, the rhizosheath weight per plant was significantly different, in which a markedly greater weight of rhizosheath per plant was found in Up1 and Zh3 than in Nip and Dj (Fig. 1D, E). This difference was due to longer roots in Up1 and Zh3 than in Nip and Dj (Fig. 1E). These results suggest that roots provide the location for rhizosheath formation and that root length is an important factor in rhizosheath formation. On the other hand, when the root length was similar in the root hair mutants (short root hair) and WT rice (long root hairs), the rhizosheath weight per plant was higher in WT than in the root hair mutants (Fig. 3E). Thus, both root hair length and root length are important in rhizosheath formation.

Transcriptome and RT-qPCR results showed that genes belonging to the ABA, auxin, root growth, and root hair pathways were regulated under MWS in rhizosheath roots (root bound with rhizosheath) of Nip and Up1 (Fig. 4). Previous studies have demonstrated that interactions between ABA and auxin play critical roles in root development and environmental responses (Du *et al.*, 2013; Xu *et al.*, 2013). ABA enhances auxin signaling by activating auxin-responsive promoters (Zhao *et al.*, 2014), while auxin homeostasis influences ABA synthesis, and the balance between auxin and ABA homeostasis is important in response to water stress (Shi *et al.*, 2014). Moreover, auxin is required for the initiation and continuation of root-hair growth (Jones *et al.*, 2009) and promotes root growth by increasing meristem size (Dello Ioio *et al.*, 2008). Though there were many genes that were similarly regulated in both Nip and Up1 rice, both root length and average root hair length were increased under MWS in Up1 rice, while only average root hair length was promoted under MWS in Nip rice (Fig. 1B; Supplementary Fig. S3A). These differences may due to comprehensive regulation of multiple genes related to ABA, auxin, and root development pathways. Because root length and root hair length are critical in rhizosheath formation, ABA and auxin pathways may be involved in rhizosheath formation via promoting root and root hair growth under MWS.

Additionally, we found that under MWS, the root hair length was significantly longer in WT rice than in the shortest root-hair mutant rice, *rhl1-1*, resulting in more rhizosheath in WT rice than that in *rhl1-1* rice. Consistently, root basipetal auxin transport and root exogenous ABA content were higher in WT rice than in the shortest root-hair mutant, *rhl1-1* (Fig. 5). However, the expression of the gene *Rab16D* (ABA gene) and the gene *PIN5B* (auxin gene) was greatly up-regulated in *rhl1-1* under the MWS condition compared with the WT (Fig. 4). It was reported that *Rab16D* only modulates the ABA responsive signaling pathway that regulates the downstream stress-responsive gene without altering the ABA biosynthesis pathway under drought stress (Tiwari *et al.*, 2019). Compared with WT rice, low ABA content in *rhl1-1* rice may require a higher ABA response under MWS. In rice plants, *PIN5B* is important for auxin homeostasis, and the paper by Lu *et al.* (2015) reported that although IAA content was increased in overexpression-PIN5b lines, the capacity of auxin transport was decreased compared with WT rice plants. Compared with WT rice, low auxin transport in *rhl1-1* rice may require a higher auxin homeostasis. These results suggest that root hair length greatly influences rhizosheath formation based on similar genetic backgrounds, and ABA and auxin might be involved in this process. When ABA biosynthesis was inhibited under MWS with fluridone (an ABA biosynthetic inhibitor), the root ABA content and root basipetal auxin transport were decreased, resulting in shorter root hair length and lower rhizosheath weight per plant in both WT rice and *rhl1-1* rice (Fig. 5). However, WT rice had a higher root endogenous ABA content, root basipetal auxin transport, average root hair length, and amount of rhizosheath per plant than *rhl1-1* under MWS with fluridone (Fig. 5). These results suggest ABA may regulate rhizosheath formation by increasing root and root hair growth under MWS. When auxin transport was inhibited by NPA (an auxin efflux inhibitor) in WT rice and *rhl1-1* rice under MWS, root ABA content did not change and root basipetal auxin transport was suppressed, resulting in lower average root hair length and weight of rhizosheath both in WT rice and *rhl1-1* rice. However, WT rice still had a higher root basipetal auxin transport, average root hair length, and amount of rhizosheath per plant than *rhl1-1* rice under MWS with NPA (Fig. 5). These results suggested that auxin may also regulate rhizosheath formation via root and root hair under MWS. Moreover, when ABA biosynthesis was inhibited under MWS, auxin transport was decreased; besides, when auxin transport was inhibition under MWS, root ABA content did not change; thus, based on our previous paper (Xu *et al.*, 2013) and these results (Fig. 5), ABA might mediate auxin transport in rhizosheath formation under water stress.

**Fig. 6.**
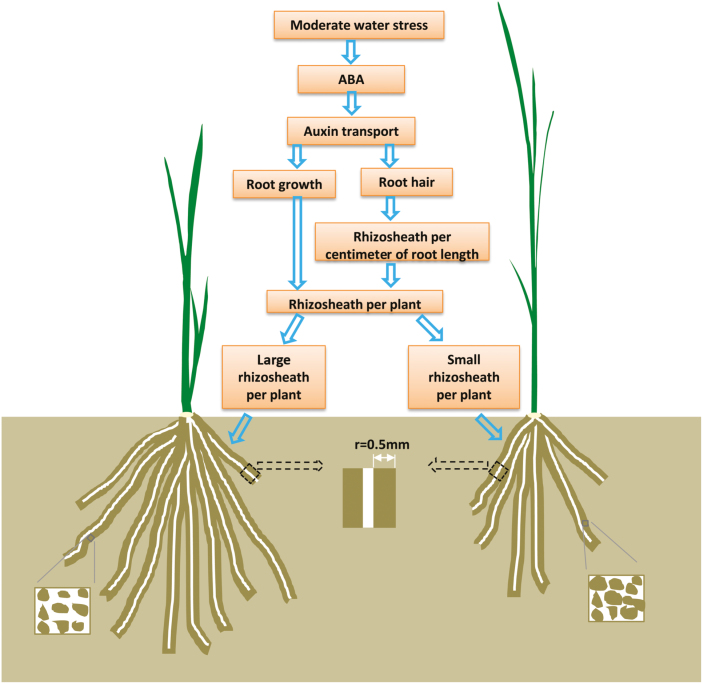
A proposed model for rice rhizosheath formation under moderate water stress. (This figure is available in color at *JXB* online.)

In conclusion, all rice varieties form rhizosheaths under MWS conditions but not under CF conditions (Fig. 6). A greater soil porosity and higher water content were found in the rhizosheaths in Up1 and Nip than in bulk soil. Different rice varieties, wild-type rice and short root hair mutants, were used to show that both root hair length and root length determined the rhizosheath weight per plant. Moreover, rhizosheath formation was regulated by the ABA, auxin, root hair and root growth pathways in response to MWS, which was subsequently verified in WT rice and the shortest root hair mutant, *rhl1-1*, by hormone analysis and pharmacological methods. Under MWS, the root exogenous ABA content, root basipetal auxin transport, and amount of rhizosheath per plant were higher in WT rice than in *rhl1-1*. Further, when ABA biosynthesis and auxin efflux were blocked in WT rice and *rhl1-1*, rhizosheath formation was significantly decreased due to shorter root hair length under MWS; however, WT maintained a higher root ABA content, higher root basipetal auxin transport, longer root hair length, and more rhizosheath than *rhl1-1*. Therefore, rhizosheath formation under moderate water stress is mediated by the ABA and auxin responses in regulating root and root hair growth (Fig. 6). This trait may be used to breed rice varieties resistant to drought, which is important for sustainable agriculture.

## Supplementary data

Supplementary data are available at *JXB* online.

Dataset S1. GO enrichment analysis of differentially expressed genes.

Dataset S2. Differentially expressed genes related to the ABA response, auxin response, and root and root hair growth in Nip rice.

Dataset S3. Differentially expressed genes related to the ABA response, auxin response, and root and root hair growth in Up1 rice.

Fig. S1. Root length of Nip rice under continuous flooding, moderate water stress, and severe water stress conditions.

Fig. S2. Above-ground traits of four rice varieties (Nip, Dj, Up1, and Zh3) under moderate water stress and continuous flooding conditions.

Fig. S3. Root hair response of four rice varieties (Nip, Dj, Up1, and Zh3) under moderate water stress and continuous flooding conditions.

Fig. S4. Root traits of wild-type (WT, Ka) rice and root hair mutants (*expa17* and *rhl1-1*) under moderate water stress (MWS), MWS with fluridone (FLU, an ABA biosynthetic inhibitor), or MWS with NPA (an auxin efflux inhibitor) conditions.

Fig. S5. Differentially expressed genes in the roots that were bound with rhizosheaths under MWS compared with no rhizosheath under CF in two rice varieties (Nip and Up1).

Table S1. Primers used in this study.

Table S2. Summary of the RNA-seq data.

Table S3. Fold changes in gene expression in the roots that were bound with rhizosheaths under MWS compared with no rhizosheath under CF in two rice varieties (Nip and Up1) determined by RNA-seq and RT-qPCR.

eraa021_suppl_Supplementary_Tables_S1-S3_Figures_S1-S5
