# Supplementary material for: Moderate water stress in rice induces rhizosheath formation associated with abscisic acid and auxin responses
Source: J Exp Bot. 2020 Feb 8;71(9):2740–51. doi: 10.1093/jxb/eraa021 (PMC7210750; doi:10.1093/jxb/eraa021)
Supplement: eraa021_suppl_Supplementary_Tables_S1-S3_Figures_S1-S5 [file eraa021_suppl_supplementary_tables_s1-s3_figures_s1-s5.pdf]

SUPPLEMENTARY DATA

Moderate water stress induces rice rhizosheath formation associated with ABA and auxin responses

Yingjiao Zhang[1, 2], Huan Du[1], Yao Gui[1], Feiyun Xu[1], Jianping Liu[1], Jianhua Zhang[3], Weifeng Xu[1,*]

[1] Center for Plant Water-use and Nutrition Regulation and College of Life Sciences, Joint International Research Laboratory of Water and Nutrient in Crop, Fujian Agriculture and Forestry University, Jinshan Fuzhou 350002, China

[2] Institute of Oceanography, Minjiang University, Fuzhou 350108, China

[3] Department of Biology, Hong Kong Baptist University, State Key Laboratory of Agrobiotechnology in the Chinese University of Hong Kong, 999077, Hong Kong

[*] Corresponding author: Weifeng Xu, E-mail: wfxu@fafu.edu.cn.

Table S1 Primers used in this study.

Primer	sequence (5′ → 3′)
Os11g0454200-F	GAGGGAGGAGGAAGAAGGGA
Os11g0454200-R	GCCAGTGTTCCCCATCATCT
Os11g0453900-F	GAGGAAGGAGGAAGAAGGGGA
Os11g0453900-R	GATCTTGGTATCGTGGCCCTG
Os05g0349800-F	CAGACGAGGAAGGAGCAGATG
Os05g0349800-R	TACTTGGACTCGTCGATGTCG
Os02g0445600-F	AGTTCCAGGAGCTTCTCAGGA
Os02g0445600-R	CATCTGATGGAGGTGGCAGAG
Os09g0505400-F	CTCCTGCGTCTTGCCATCATA
Os09g0505400-R	GATCAACACCGGTAATGACGC
Os04g0519700-F	CATTGGCAGCGATGAACTGAG
Os04g0519700-R	AGTATCCTAAGCTTACGCGCC
Os07g0418700-F	GAGTCCTAACCCCAAGCCAAA
Os07g0418700-R	TCAGTTCCCGGGCACAATTAT
Os07g0418600-F	GAGCCAAGTCCTAACCCCAAG
Os07g0418600-R	ATTGCTGGTGAAAGAGGTGGT
Os11g0582300-F	CTCAGGTGCAGTATCAGGCTC
Os11g0582300-R	AGGCTTCAGGATTCGTCATCG
Os01g0111500-F	ATTCCTCCAGCTGCAAGTGAA
Os01g0111500-R	AAGGCGTCCTTGACTTGAGAG
Os04g0659300-F	CATGAGCAGCACCAACATCAG
Os04g0659300-R	GAACAGCTTCCTCGCGATGTC
Actin1-F	TGGCATCTCTCAGCACATTCC
Actin1-R	TGCACAATGGATGGGCCAGA

Table S2 Summary of the RNA-seq data.

Sample	Total Raw Reads(M)	Total Clean Reads(M)	Total Clean Bases(Gb)	Clean Reads Q20(%)	Clean Reads Q30(%)	Clean Reads Ratio(%)
NipMWS_1	21.94	21.79	1.09	98.8	92.47	99.29
NipMWS_2	21.94	21.66	1.08	98.81	92.65	98.71
NipMWS_3	21.94	21.89	1.09	98.79	92.53	99.76
NipCF_1	21.94	21.87	1.09	98.77	92.4	99.68
NipCF_2	21.94	21.86	1.09	98.74	92.4	99.61
NipCF_3	21.94	21.9	1.09	98.77	92.26	99.8
Up1MWS_1	21.94	21.87	1.09	98.8	92.71	99.66
Up1MWS_2	21.94	21.82	1.09	98.77	92.4	99.43
Up1MWS_3	21.94	21.85	1.09	98.7	92.15	99.6
Up1CF_1	21.94	21.91	1.1	98.75	92.32	99.83
Up1CF_2	21.94	21.88	1.09	98.8	92.5	99.72
Up1CF_3	21.94	21.89	1.09	98.81	92.57	99.78

Table S3 Fold changes in gene expression in the roots that were bound with rhizosheaths under MWS compared with no rhizosheath under CF in two rice varieties (Nip and Up1) determined by RNA-seq and RT-qPCR.

		RNA-seq (log_2Ratio)		RT-qPCR (log_2Ratio)	
Symbol	Annotation[a]	Up1[b]	Nip[b]	Up1[b]	Nip
Os11g0454200	dehydrin Rab16B	7.80	9.33	2.38	4.29 [b]
Os11g0453900	dehydrin Rab16D	8.23	7.71	2.02	3.69 [b]
Os05g0349800	embryonic abundant protein 1(Emp1)	6.63	6.54	4.68	2.95 [b]
Os02g0445600	auxin-induced protein 15A	1.40	1.71	1.68	0.57
Os09g0505400	probable auxin efflux carrier component 5b	1.76	1.61	1.84	1.06
Os04g0519700	auxin response factor 10	1.58	1.34	2.28	1.12
Os07g0418700	protein TsetseEP	1.40	8.84	1.91	5.39 [b]
Os07g0418600	protein TsetseEP	2.65	6.18	3.99	3.51 [b]
Os11g0582300	protein ROOT HAIR DEFECTIVE 3 homolog 2-like (RHD3)	1.43	1.79	1.54	1.22 [b]
Os01g0111500	transcription factor bHLH83	N.S.	-1.05	N.T.	-0.72
Os04g0659300	cysteine-rich receptor-like protein kinase 6(CRRS38)	-3.44	-3.41	-2.05	-1.51 [b]

Abbreviations: N.S., not significance; N.T., not tested.

[a]Annotation based on NCBI nr.

[b]Fold change value with statistical significance (P<0.05).

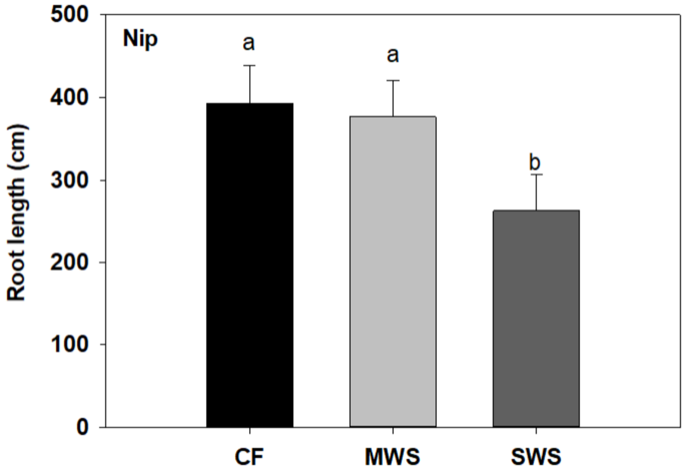

Fig. S1. Root length of Nip rice under continuous flooding (CF), moderate water stress (MWS) and severe water stress (SWS) conditions. Data are the means ± SE of four replicates. The bars with different letters were significantly different at $P<0.05$.

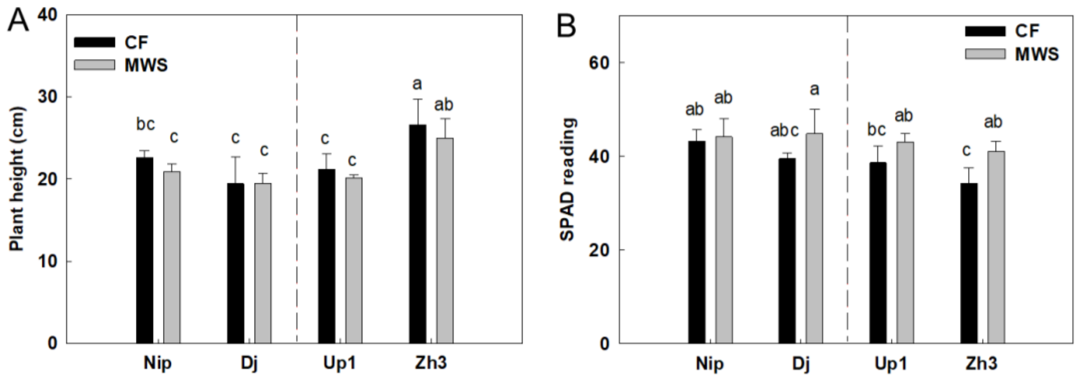

Fig. S2. Above-ground traits of four rice varieties (Nip, Dj, Up1 and Zh3) under moderate water stress (MWS) and continuous flooding (CF) conditions. (A) Plant height. (B) Leaf chlorophyll content. Data are the means ± SE of four replicates. The bars with different letters were significantly different at $P<0.05$.

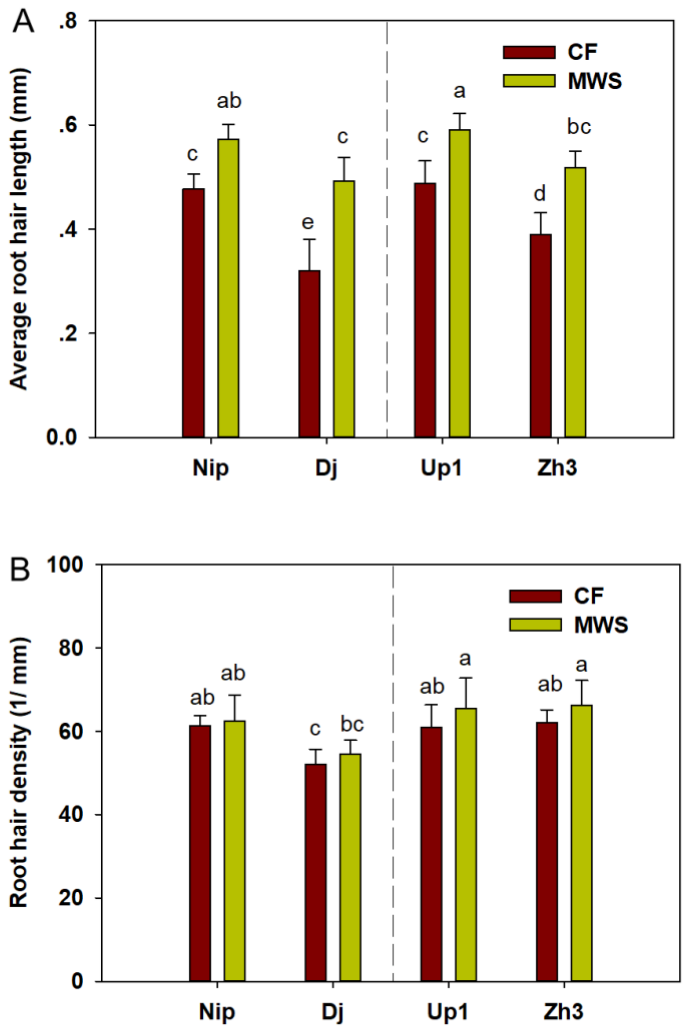

Fig. S3. Root-hair response of four rice varieties (Nip, Dj, Up1 and Zh3) under moderate water stress (MWS) and continuous flooding (CF) conditions. (A) Average root hair length. (B) Root-hair density. Data are the means ± SE of four replicates. The bars with different letters were significantly different at $P<0.05$.

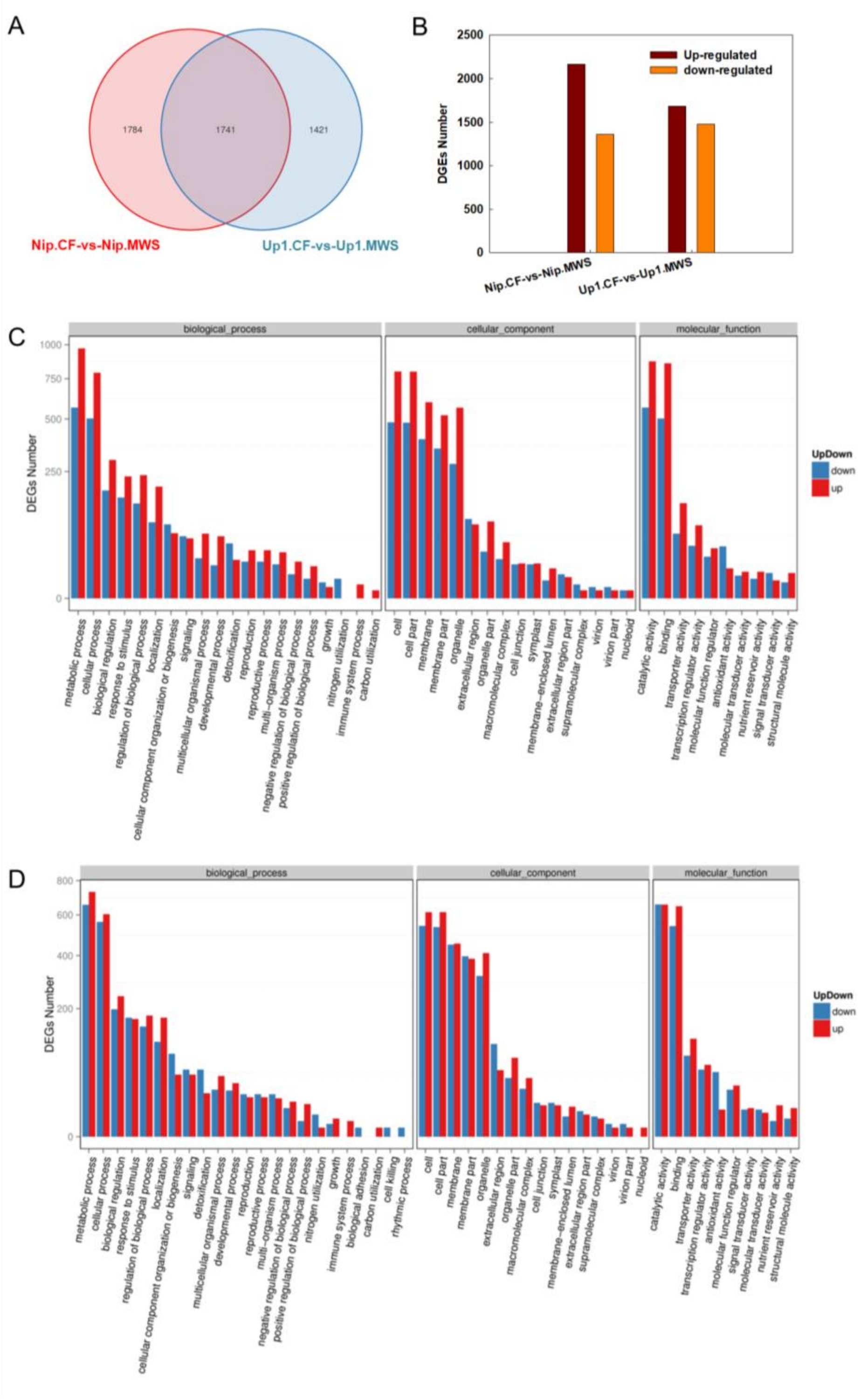

Fig. S4 Differentially expressed genes (DEGs) in the roots that were bound with rhizosheaths under MWS compared with no rhizosheath under CF in two rice varieties (Nip and Up1). (A) Venn diagram of the DEGs in Nip and Up1. (B) Numbers of upregulated and downregulated DEGs in Nip and Up1. (C) Number of DEGs associated with the most enriched GO terms in (C) Nip and (D) Up1.

Fig. S5 Root traits of wild-type (WT, Ka) rice and root hair mutants (*expa17* and *rhl1-1*) under moderate water stress (MWS), MWS with fluridone (FLU, an ABA biosynthetic inhibitor) or MWS with NPA (an auxin efflux inhibitor) conditions. (A) The root length was not significantly different in WT rice, *expa17* (shorter root hair mutant) and *rhl1-1* (the shortest root hair mutant). (B) There were no differences in the root fresh weight, (C) root dry weight, (D) water content of the root and (E) root length between WT and *rhl1-1* under MWS, MWS with FLU, and MWS with NPA. Data are the means ± SE of four replicates. The bars with different letters were significantly different at $P<0.05$.

Supplemental Dataset 1. GO enrichment analysis of differentially expressed genes (DEGs, Excel file separately attached).

Supplemental Dataset 2. Differentially expressed genes (DEGs) related to the ABA response, auxin response, root and root hair growth in Nip rice. (Excel file separately attached).

Supplemental Dataset 3. Differentially expressed genes (DEGs) related to the ABA response, auxin response, root and root hair growth in Up1 rice. (Excel file separately attached).
